# Factors Influencing Recurrence after Surgical Treatment of Odontogenic Maxillary Sinusitis: An Analysis from the Oral and Maxillofacial Surgery Point of View

**DOI:** 10.3390/jcm12113670

**Published:** 2023-05-25

**Authors:** Andreas Sakkas, Christel Weiß, Marcel Ebeling, Sebastian Pietzka, Frank Wilde, Theo Evers, Oliver Christian Thiele, Robert Andreas Mischkowski, Mario Scheurer

**Affiliations:** 1 Department of Cranio-Maxillo-Facial-Surgery, University Hospital Ulm, 89081 Ulm, Germany; 2 Department of Cranio-Maxillo-Facial-Surgery, German Armed Forces Hospital Ulm, 89081 Ulm, Germany; 3 Medical Statistics and Biomathematics, University Medical Centre Mannheim, Heidelberg University, 68167 Mannheim, Germany; 4 Department of Otolaryngology, Head and Neck Surgery, German Armed Forces Hospital Ulm, 89081 Ulm, Germany; 5 Department of Cranio-Maxillo-Facial-Surgery, Ludwigshafen Hospital, 67063 Ludwigshafen, Germany

**Keywords:** Bichat’s fat pad, infection, maxillary sinus, recurrence, risk factors, sinusitis

## Abstract

The aim of the study was to determine the factors influencing the development of recurrence after the surgical treatment of odontogenic maxillary sinusitis in an oral and maxillofacial surgery clinic over a 7-year period. Demographic and anamnesis data, clinical and radiological findings, treatment and outcome were analyzed. A multivariable analysis was performed to find associations between patient age, causative focus, surgical access for sinus revision, multilayer closure with a buccal fat pad, inferior meatal antrostomy (IMA) for temporary sinus drainage and sinusitis recurrence. A total of 164 patients with a mean age of 51.7 years were included. Sinusitis recurrence was observed in nine patients (5.48%) within 6 months after primary surgery. No significant correlation was detected between patient age, causative focus, surgical access for sinus revision, multilayer closure with a buccal fat pad, IMA for sinus drainage and the development of recurrence (*p* > 0.05). Patients with a history of antiresorptive-related osteonecrosis of the jaw showed a significant tendency toward disease recurrence (*p* = 0.0375). In conclusion, except for antiresorptive administration, none of the investigated variables were related to a higher risk of sinusitis recurrence. We encourage a combined approach of intraoral removal of the infective focus and sinus drainage via FESS, as well as an individual treatment decision in a multidisciplinary setting with collaboration between dentistry, maxillofacial surgery and otorhinolaryngology to avoid sinusitis recurrence.

## 1. Introduction

Odontogenic maxillary sinusitis is one of the most common diseases in the fields of otolaryngology, maxillofacial surgery and dentistry [1,2,3,4]. It encompasses diseases characterized by inflammatory or infectious processes in the environment of the maxillary sinus as a result of odontogenic sources [5]. Rhinogenic sinusitis, with a rate of approximately 68%, has to be separated, since its etiology is different and mostly occurs bilaterally [6,7,8]. The literature states that about 10–41% of all sinusitis cases are triggered by odontogenic pathology [9,10,11,12,13,14].

Considering the first description of this condition, despite the fact that the term odontogenic maxillary sinusitis strictly refers to the inflammatory process of the maxillary sinuses due to dentogenic foci, the contemporary scientific literature has expanded the term by including other potential causes [15]. Consequently, odontogenic sinusitis covers all paranasal sinus infections caused by dental disease (e.g., chronic apical periodontitis), and also dental procedures (e.g., extractions, endodontic treatments), oral surgery procedures (e.g., dental implantations) and antiresorptive-related osteonecrosis of the jaw (ARONJ) or osteomyelitis of different origin [2,3,16,17]. In particular, sinus floor elevation and grafting with autologous, synthetic or deproteinized bovine bone for implantological purposes can affect the homeostasis of the maxillary sinus. The absence of sinusitis symptoms is mandatory prior to this surgical procedure and postoperative maxillary sinusitis is considered as a common complication [18,19,20]. Oroantral fistula, as result of unnatural communication between the oral cavity and maxillary sinus with epithelialization in the fistula tract, has a rate of approximately 60% and is also among the most common causes of odontogenic sinusitis [9,17,21].

The appropriate diagnosis and treatment of odontogenic maxillary sinusitis has represented a common ground for otolaryngologists and maxillofacial surgeons [22,23,24]. A recent multidisciplinary consensus statement addressed some controversial issues regarding the appropriate therapy plan after a revision of the scientific literature [1]. However, concerning the optimal sequence of surgical interventions, the majority of contemporary otorhinolaryngologic literature recommends primarily addressing the odontogenic infection source [10,15,23]. Other authors suggest a combined approach of oral removal of dental infection and simultaneously maxillary sinus drainage via endoscopic sinus surgery (FESS) [9,25,26,27]. The need for a two-discipline approach depends on the mucociliary transport, anatomy of the sinus ostium and the necessary width of the access to the maxillary sinus [4,26].

Despite the currently developed function-preserving surgical interventions, sinusitis recurrence is a clinical challenge for treating physicians of the involved disciplines and the causes for persistent disease have still not been clearly investigated. Galli et al. proposed diabetes mellitus and nicotine consumption as potential risk factors for disease recurrence [9]. Another work stated that patients with a delayed diagnosis of odontogenic source sinusitis have a higher possibility of surgical revision after primary intervention [10]. To the best of our knowledge, no clear evidence-based literature exists regarding specific factors influencing the rate of recurrence after the surgical treatment of odontogenic maxillary sinusitis.

The aim of the study was to assess the incidence and to determine the factors influencing the development of recurrence of odontogenic maxillary sinusitis after surgical treatment in an oral and maxillofacial surgery unit. Thus, we aimed to improve the surgical outcome by specifying demographic and surgical factors as predictors of a higher incidence of disease recurrence from a maxillofacial surgery point of view.

## 2. Materials and Methods

### 2.1. Patient Cohort

For this observational retrospective single-center study, we reviewed the medical records of all patients with unilateral odontogenic maxillary sinusitis who were surgically treated in our clinic of oral and plastic maxillofacial surgery between January 2015 and August 2022. The records were retrieved from our hospital electronic database. Ethical approval for this study was obtained from the Ethics Committee of the Chamber of Physicians in Rhineland-Palatine, Mainz, Germany (approval number: 2022-16464; approval date: 21 April 2022), and the study was performed in accordance with the Declaration of Helsinki 1964 and its later amendments (World Medical Association, Declaration of Helsinki).

We enrolled patients who fulfilled the following inclusion criteria: (1) full range of age, (2) clinically and radiologically confirmed unilateral maxillary sinusitis of odontogenic or other intraoral origin, and (3) patients who underwent surgical treatment via an intraoral approach. Exclusion criteria were (1) patients with diagnosed rhinogenic sinusitis, (2) patients who received conservative symptomatic treatment, and (3) patients with incomplete medical charts.

### 2.2. Patient Screening

The diagnosis of odontogenic maxillary sinusitis was based on anamnestic data of sinusitis-related symptoms, clinical assessment and radiological imaging. Clinical symptoms included the presence of nasal obstruction, increased purulent nasal secretion, facial pain, tender on pressure in the lateral midface, dentogenic bite sensitivity and other nonspecific general symptoms. Our standard radiological protocol for diagnosis included panoramic radiographs and a CT or CBCT scan to assess the causative focus, the extent of disease and the anatomic features such as sinus septum and natural ostium. Board-certified oral and maxillofacial surgeons indicated surgical sinus revision after an evaluation of the clinical and radiological findings.

Surgery was performed under intubation anesthesia in all cases. A single shot of 2/1 gr ampicillin/sulbactam (Unacid^®^, Pfizer Pharma GmbH, Berlin, Germany) or, if penicillin allergic, 600 mg clindamycin (Clinda-saar^®^, MIP Pharma GmbH, Blieskastel, Germany) as well as 250 mg prednisolone (Solu-Decortin^®^, Merck Pharma GmbH, Weiterstadt, Germany) was administered intravenously to patients preoperatively. The surgical method used was based on the original cause of sinusitis and the radiologic extent of the disease.

Depending on the causative focus and its localization, access to the maxillary sinus for revision was performed through (1) transantral fenestration of the anterior sinus wall using the bone lid method (elevation of bone fragment from the facial wall of the maxillary sinus), according to Lindorf [28], (2) transantral fenestration of the anterior sinus wall via a drill hole, or (3) transalveolarly when sufficient access with direct communication of the oral and sinus mucosa was present after focus removal. In each case, surgical maxillary sinus revision was performed by removing the intrasinusal pathologic material. Postoperatively, all patients received appropriate analgesics and nasal decongestive drops and were advised to perform frequent saline irrigations and avoid sniffing for two weeks after surgery. Control panoramic radiographs were performed directly post-operation.

All patients were followed up for a minimum of 6 months postoperatively to assess the outcome of sinus surgery. Patients with signs of clinical improvement and regression of the preoperative sinusitis symptoms were defined as successfully treated. Sinusitis recurrence was defined as the reappearance of sinusitis-related symptoms and the presence of radiological signs of the disease within 6 months after primary surgery. Repeat imaging was performed only in cases of suspected disease recurrence and not on a regular basis.

### 2.3. Data Collection

Data were collected from patients’ electronic hospital charts and patients were anonymized before data analysis. Extracted data comprised patient age, gender, sinusitis-related symptoms, intraoral causative focus, surgical treatment for focus elimination, surgical access to the maxillary sinus, multilayer soft tissue closure of an oroantral communication using the buccal fat pad (Bichat’s fat pad), performance of inferior meatal antrostomy (IMA) for temporary sinus drainage, the duration of hospitalization in days and rate of disease recurrence.

We collected all CT and CBCT scan prescriptions provided by the attending clinician after the clinical evaluation. All radiologic scans followed the standard institute protocol and were interpreted by two board-certified radiologists. We extracted all radiological findings that were causatively relevant to the development of maxillary sinusitis. The exact causative focus for the disease was extracted from the radiological and operation reports.

### 2.4. Statistical Analysis

Data were centralized in an electronic format using Microsoft Excel software and were analyzed descriptively. Statistical analysis was performed using SAS^®^, Release 9.4 software (SAS Institute Inc., Cary, NC, USA). Descriptive statistics were used to describe baseline patient and surgery characteristics. All categorical variables were expressed as absolute values (n) and relative incidence (%). Patient age was presented with the mean value and standard deviation. A multivariable analysis was performed to find associations between the possible influencing variables (patient age, causative focus, surgical access for sinus revision, multilayer closure with a buccal fat pad, IMA for sinus drainage) and the development of sinusitis recurrence. Associations between categorical variables were described by cross-tabulations, and Fisher’s exact tests were used to investigate a potential association between the variables and disease recurrence. To compare the length of hospitalization in patients who received an IMA and those who did not, the Mann–Whitney U test was used. Prior to that, the verification of data normality with the Kolmogorov–Smirnov test showed an abnormal distribution. The length of hospitalization was represented by a median value (in days) and the maximum–minimum (dispersion measure). A two-sided *p* value of less than 0.05 was considered statistically significant. The results were presented as tables and bar charts.

## 3. Results

### 3.1. Demographic Distribution

A total of 164 patients were included in the analysis. There were more males (84/164; 51.2%) than females (80/164; 48.8%) and the male–female ratio was 1.05:1. The patient age at the time of surgery ranged from 11 to 86 years, with a mean ± SD age of 51.7 ± 17.7 years. Most of the patients (71.3%) were younger than 65 years. The patients’ overall baseline characteristics are presented in Table 1.

### 3.2. Sinusitis Symptoms and Causative Focus of Odontogenic Maxillary Sinusitis

Ninety-nine patients (60.4%) reported sinusitis complaints at the initial clinical examination. The most common orodental etiology for maxillary sinus infection was chronic apical periodontitis (*n* = 85; 51.8%), with 81.2% of cases diagnosed in the molars and 18.8% in the premolars of the maxilla. Oroantral communication was diagnosed as infective focus in 26.8% (*n* = 44) of the cases, and, among them, 15.9% (*n* = 7) were after the osteotomy of premolars and 84.1% (*n* = 37) were after the osteotomy of molars, respectively. Antiresorptive-related osteonecrosis of the jaw was documented in 9.1% (*n* = 15) of the included patients; among them, there were 12 cases with a history of bisphosphonate therapy and 3 cases with a history of desonumab therapy, respectively. The distribution of sinusitis etiology is presented in Table 1.

### 3.3. Surgical Treatment

Considering the osteoplastic access to the maxillary sinus, the transalveolar approach was the most commonly used in 53.7% (*n* = 88) of the cases. This approach was most commonly used in cases of oroantral communication (*n* = 32/44; 72.7%), in 47.1% (*n* = 40/85) of CAP cases, and in 86.7% (*n* = 13/15) of ARONJ cases. Fenestration of the anterior maxillary sinus wall with the bone lid method was performed in 41.2% (*n* = 69/164) and fenestration via a burr hole was performed in 4.3% (*n* = 7/164) of the study cases, respectively. These two methods were used non-specifically, independent of the causative focus, but always in cases of antrally displaced foreign bodies and odontogenic cysts due to the better intrasinusal visualization.

Multilayer surgical closure with a pedicled buccal fat flap was performed in 41 patients (25%); among them, in thirty-four cases of oroantral communication, there were six cases of decortication by ARONJ and one case of explantation by peri-implantitis.

An inferior meatal antrostomy for sinus drainage was performed in 43.9% (*n* = 72/164) of the patients (Table 1).

### 3.4. Postoperative Outcome

In total, the median post-operative length of hospitalization was 3 days (minimum = 0, maximum = 12). The length of hospitalization was significantly longer for patients who received an IMA (median = 4 days; minimum = 0, maximum = 11) than for those without an IMA (median = 2 days; minimum = 0, maximum = 12) (Mann–Whitney U test: *p* < 0.0001).

Sinusitis recurrence was diagnosed in nine patients (5.48%) within 6 months after primary surgery. Regarding the initial causative focus for maxillary sinusitis in these patients, three had an oroantral communication, three an ARONJ, two a CAP and one had osteomyelitis. In these patients, initial surgical access for sinus revision was performed transalveolarly in six, and through transantral fenestration with the bone lid method in three cases, respectively.

### 3.5. Multivariable Analysis

The multivariable analysis revealed no significant correlation between patient age, causative focus, surgical access for sinus revision, multilayer closure with a buccal fat pad, and intraoperative IMA for sinus drainage and the development of sinusitis recurrence.

Regarding the etiology, no correlation could be detected between the causative focus in general and sinusitis recurrence (Fisher’s exact test: 0.1046). However, the subgroup analysis showed a significantly higher tendency for sinusitis recurrence in cases of ARONJ (Fisher’s exact test: *p* = 0.0375) (Table 2).

Among the 117 patients younger than 65 years, disease recurrence occurred in 6 of them (5.13%). On the contrary, disease recurrence was diagnosed in 6.38% (*n* = 3) of the 47 patients older than 65 years. No significant association between patient age and sinusitis recurrence was detected (Fisher’s exact test: *p* = 0.7166) (Table 3).

In eight out of nine recurrence cases, multilayer closure with the buccal fat pad was not performed. Regarding the multilayer closure with a buccal fat pad, no association with the development of sinusitis recurrence could be detected (Fisher’s exact test: *p* = 0.4526) (Table 3).

Regarding the intraoperative sinus drainage via IMA, no correlation with the development of sinusitis recurrence could be detected (Fisher’s exact test: *p* = 0.3010). IMA was performed in seven out of nine patients with disease recurrence (Table 3).

**Figure 1:**
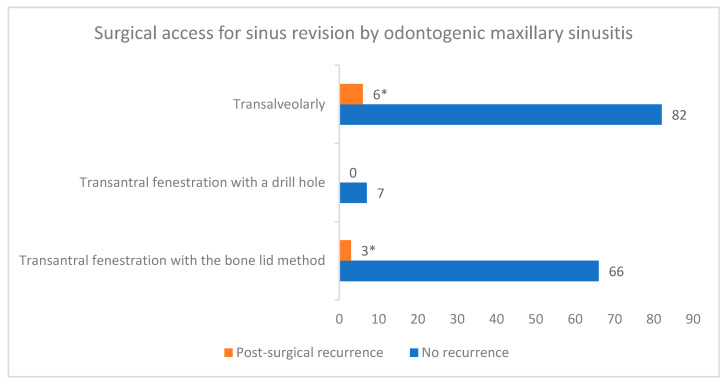
Correlation between surgical access for sinus revision and recurrence of odontogenic maxillary sinusitis. * Fisher’s exact test: *p* = 0.8209.

Regarding the surgical access for sinus revision, no association could be detected between the three surgical approaches used in this cohort and the incidence of sinusitis recurrence (Fisher’s exact test: *p* = 0.8209). In particular, transalveolar access led to recurrence in six cases and transantral fenestration with the bone lid method in three cases, respectively (Figure 1).

## 4. Discussion

Odontogenic maxillary sinusitis is a common pathologic condition that must be addressed in an interdisciplinary manner by specialists in the fields of otolaryngology, oral and maxillofacial surgery and dentistry to ensure sufficient treatment and the avoidance of recurrence or chronicity. We aimed to specify demographic and surgical factors as predictors of a higher incidence of disease recurrence. Our results provide valuable insights into the treatment of these patients from a maxillofacial surgery point of view, addressing the lack of an evidence base in relation to this topic.

Most of the studies regarding odontogenic maxillary sinusitis differ in methodology, design and disease definition which limits their comparability. Our study represents a considerable cohort that is commonly seen internationally within the same period. Considering the demographic distribution, the much-reported female predominance could not be reproduced in this study [4,17,27,29]. The mean age of our cohort was 51.7 years and thus was in line with previously published data reporting an older mean age [27,29]. Only 60.4% of our cohort presented clinical symptoms compatible with therapy-resistant chronic unilateral sinusitis, confirming the high proportion of asymptomatic patients with accidental sinusitis-related CT findings described in the study of Naros et al. [27].

Our results present periapical pathology as the most common etiology for maxillary sinus infection. On the contrary, Tröltzsch et al. and Galli et al. reported post-surgical oroantral fistulae as the pivotal triggering factor and leading cause of odontogenic maxillary sinusitis [9,17]. Over time, there might have been a change in the etiologic factors of this disease profile. Recent scientific evidence suggests the increasing number of maxillofacial surgery procedures, such as sinus floor elevations, and diseases, such as medication-related osteonecrosis or osteoradionecrosis, as additional potential causes of sinus pathology [9,16,17,18,20,22]. Regarding this, Candotto et al. and Kim et al. postulated that dental implants have a major role in the development of symptomatic maxillary sinusitis [20,30]. Our study found that only 1.8% of sinusitis cases had a history of implant surgery. However, we highlight the 9.1% incidence of ARONJ and 6.1% incidence of foreign bodies as upcoming infection causes and recommend that a careful medical history is taken in specific patient groups. Tröltzsch et al. reported that the incidence of medication-related osteonecrosis of the jaw was 4.5% in a study of 174 patients [17]. Considering our study results, which included various potential causes, we recommend the modification of the term “odontogenic” to “orodental” to describe maxillary sinusitis with multifactorial intraoral etiology.

We showed a recurrence rate of 5.5% of odontogenic maxillary sinusitis after the elimination of the infectious focus and sinus revision. The incidence of recurrence varies in the literature, depending on the type of study and patient cohort. Galli et al. presented comparable results with a recurrence rate of 5.9% in a cohort of 34 patients; however, unlike our study, all patients were treated with combined FESS and oral surgery [9]. Naros et al. reported one recurrence case (4.5%) among twenty-two patients with an intrasinusal fungus ball [27]. Zirk et al. documented two surgical revisions among 121 operated patients (1.6%) [4]. Molteni et al. reported a recurrence rate of less than 1% after evaluating 480 patients [31]. In general, a comparison with previous data was limited because of the different treatment protocols and the different factors investigated as recurrence triggers. For example, Galli et al. proposed diabetes mellitus and nicotine consumption as potential risk factors for disease recurrence [9]. The same researchers stated that tobacco use induces the release of catecholamines, which favors peripheral vasoconstriction with tissue ischemia and delayed healing, and, similarly, patients with diabetes mellitus have a higher tendency for postoperative complications due to having greater susceptibility to chronic inflammation [9]. In our study, we did not aim to investigate the general medical condition and smoking habit as potential influencing factors and thus future studies should focus on these.

The age limit of 65 years was not found to be associated with disease recurrence. We could assume that the mucociliary clearance through the natural sinus mucosa could be disturbed with increasing age, resulting in a higher rate of recurrence; however, this was not confirmed by our results and no comparison with the present literature was possible. Therefore, the impact of age on the development of sinusitis recurrence has to be investigated in future studies.

Considering the disease etiology, patients with ARONJ had a particular, statistically significant tendency to develop recurrent odontogenic sinusitis in the overall population studied. This was the only cause with a significant impact on recurrence in our data. This finding in this patient cohort could be explained due to the overall reduced immune defense and the local anti-angiogenic effect that could lead to an uneventful healing process and the extension of the alveolar pathology into the maxillary sinus. Our assumption is consistent with the retrospective case study of Maurer et al. who diagnosed odontogenic maxillary sinusitis in 48% of patients with bisphosphonate-associated osteonecrosis of the jaw in the maxillary region [32]. None of our ARONJ patients showed evidence of maxillary sinus infection with, e.g., actinomycetes or fungi, which could possibly be due to perioperative, prolonged intravenous antibiotic therapy, which was also discussed by Maurer et al. [32]. Taking this into consideration, perioperative antibiotic therapy may have contributed to the prevention of sinusitis recurrence in our ARONJ patients. Considering our findings, we recommend a particularly comprehensive procedure consisting of prolonged antibiotic therapy, subtle eradication of the infectious, necrotic focus with interdisciplinary, endoscopic procurement of sufficient nasal drainage in order to ensure a minimal risk of disease recurrence in this patient group. These preliminary results have to be validated in further studies with larger numbers of patients with a history of ARONJ.

We did not detect an association between the surgical access for sinus revision and disease recurrence. Although, of note in our study was the recurrence incidence of 6.8% in patients who had received sinus revision transalveolarly, which was higher than with the other two surgical accesses used. This difference was, however, not statistically significant. In our opinion, infected and pathologic endosinusal tissues (e.g., mucoceles, cysts, granulation tissue) as well as foreign bodies cannot be adequately identified and subtly removed using transalveolar access, due to the limited intrasinusal overview of this minimally invasive method. Thus, we recommend this surgical access only in cases of infected sinuses limited to the basal sinus area, for example, in cases of small periapical pathologies without intraoperative oroantral communication. Additionally, in these cases, a multilayer wound closure with a buccal flap is of great importance in order to avoid chronic oroantral fistulae. We showed an even lower recurrence incidence after transantral fenestration with the bone lid method, and this could be explained due to the better intrasinusal visualization enabling the simultaneous insertion of a 4 mm endoscope and an additional instrument, e.g., a Blakesley forceps [27]. This minimally invasive endoscopically assisted osteoplastic approach via the anterior maxillary wall offers the opportunity for an uncomplicated, complete removal of any intrasinusal pathology and additionally allows for simultaneous treatment of the potential odontogenic origin [27,29]. The bone lid method also allows for precise repositioning of the bone fragment without remaining bony defects in the anterior sinus wall. Maurer et al. obtained similar conclusions, as they observed no differences in recurrence rates in patients with and without antral fenestration, and considered other causative factors contributing to refractory odontogenic maxillary sinusitis [32]. We also documented no difference regarding recurrence in transantral fenestration cases via the bone lid method or via a burr hole. No existing literature can validate this statement; however, we believe that a burr hole at the antral sinus wall, which provides a sufficient endosinusal overview and subsequent coverage of the bony defect with a collagen membrane, can be as successful as the bone lid method. We also find the burr hole method to be less invasive and more time efficient than the bone lid method; however, it cannot be indicated in cases of extended sinus pathology or in cases of foreign bodies. In addition, the remaining bony defect can lead to secondary infections due to possible recurring oroantral communication. In general, we consider both approaches as being minimally invasive with very low patient morbidity and the treatment decision has to be made on an individual basis. The validation of our preliminary results, regarding the more effective intraoral surgical access for sinus revision, in further prospective studies could provide valuable clinical insights for practitioners.

Regarding soft tissue closure after intraoral sinus revision, sinusitis recurrence occurred in 2.4% and 6.5% of the patients with and without multilayer closure with a Bichat fat flap, respectively. However, this difference was not significant, which might be due to the small total case number and the group inhomogeneity. Besides the eradication of the infectious focus, the second crucial cornerstone for successful therapy is the sufficient soft tissue closure in conjunction with concomitant antibiotic therapy and assurance of sufficient nasal drainage. In our study, patients with oroantral communication were treated with a buccal advancement flap. While some authors propose that small oroantral communications might heal spontaneously after adequate endoscopic treatment of the sinusitis, we prefer to perform surgical closure, no matter the size of the defect, to promote healing. The Bichat fat flap is commonly used in cases of extended oroantral communications to avoid suture dehiscence and secondary wound healing complications, especially in cases when a tension-free surgical closure is required, e.g., in cases of patients taking antiresorptive medication [33]. A prerequisite for the surgical closure of an oroantral communication is primarily to ensure sufficient drainage via the ethmoidal infundibulum, allowing the spontaneous healing of the sinusitis. If this is given, the Bichat flap is recommended to ensure multilayer wound closure after the elimination of the dentogenic focus, especially in recurrent and large defects (>5 mm) [2,21]. Parvini et al. stated that, basically, defects larger than 5 mm and existing for longer than 3 weeks should be surgically closed in a monolayer manner. The same authors recommend the multilayer closure only if primary closure is not sufficient, especially when sufficient vestibular height is available, to allow for adequate secondary prosthetic restoration [34]. In particular, regarding the prevention of recurrence due to persistent oroantral communication, multilayer closure with a Bichat fat pad seems to be particularly suitable due to its high success rate and its technically simple procedure, as Park et al. have already shown in their retrospective study [35]. We believe that a safe primary soft tissue closure can reduce the risk of persistent oroantral communication, and, thus, the risk of sinusitis recurrence. Although no data exist in the current literature regarding the impact of multilayer closure on sinusitis recurrence rate, considering our findings, we recommend the use of a Bichat fat pad due to its great versatility, easy mobilizability, vessel-targeted nutrition and low complication rate [33].

Surgical success also largely depends on the simultaneous recovery of normal sinus function through spontaneous drainage from the natural ostium [9]. In this sense, the basic principles of drainage of the involved sinuses via the ostiomeatal complex must be a central component of the therapeutic algorithm. In our study, we performed an IMA for temporary sinus drainage in almost half of the cohort. These patients presented an active sinus infection with purulent discharge intraoperatively. It was interesting that out of the nine recurrent cases, there were seven cases for which no IMA was performed, which, however, was without significance due to the small case number. The lack of similar research makes our findings incomparable, and, thus, further studies have to investigate our clinical experience. In principle, sufficient drainage is necessary in infected maxillary sinuses if the natural drainage via the sinus ostium cannot be ensured due to the inflammation-related swelling of the sinus mucosa. In cases of sufficient drainage via the middle nasal meatus, which can be checked, e.g., by minimally invasive endoscopy, IMA is not necessary because it might obstruct secretion outflow. The IMA theoretically allows for the drainage of reaccumulated material and facilitates a suction toilet after surgery [12,13]. Al-Belasy et al. also emphasized the lack of necessity to perform an IMA in patients with an open osteomeatal complex without anatomical deformities [36]. In addition, some rare but possible complications, such as iatrogenic injury to the sphenopalatine artery, which occurred in one of our cases, injury of the nasolacrimal duct with duct obstruction and epiphora, and disruption of physiology with unchanged ostially-directed mucociliary beat, have been related to IMA [36,37,38,39]. According to the literature, IMA is nowadays no longer standard in the treatment of odontogenic maxillary sinusitis and is reserved only for a few specific indications with additional middle meatotomy, for example, to gain additional access to the anterior-inferior part of the maxillary sinus in cases of extended fungus balls [40,41,42]. In addition, the combination of a middle and inferior meatotomy is supposed to obstruct the physiological mucociliary clearance, potentially resulting in a circular flow [27]. Currently, due to the widespread use of endoscopic technology, the endoscopically assisted sinus surgery (FESS) is the gold standard for restoring physiological drainage and maxillary sinus ventilation via the meatus nasi medius, substituting the more invasive, and with long-term complications, previously used Caldwell-Luc technique [2,9,12,13,17,27,29,30,31,43,44,45,46]. FESS has also gained practical importance because it offers a simultaneous revision of the often involved ethmoid and frontal sinus. Psillas et al. suggest FESS when the osteomeatal complex is blocked and the height of the thickened mucosa is more than one half of the maxillary sinus [2]. This method is less invasive and enables direct endoscopic control and treatment, allowing for surgical “toilette” and an enlargement of the maxillary ostium to favor the rapid recovery of physiological sinus functions. Additionally, cofactors of infection, including anatomical anomalies such as concha bullosa, inferior turbinate hypertrophy or septal deviation, can be corrected [9,31]. Furthermore, an endoscopic approach allows for the exploration of the other paranasal sinuses that may also be involved in the infective process [9].

In concordance with several studies, we emphasized the importance of collaboration between different professionals when managing patients with odontogenic maxillary sinusitis as this is fundamental for the prevention of recurrence [1,2,3,4,12,13,17,29,30,31,47,48]. A combined approach is useful to eliminate dental pathologies and recover the natural sinus homeostasis. Considering the difficulty in identifying a single clinician able to manage both the odontogenic infectious source and the appropriate restoration of the sinus mucosal inflammation, we highlight the importance of a multidisciplinary approach. In our opinion, the most important influencing factor for disease recurrence is a monodisciplinary treatment, which could fail both in regard to correct diagnosis and appropriate treatment, being either surgical or not. In this sense, otolaryngologists are usually more confident with the performance of FESS for limiting maxillary mucosal inflammation but could struggle in identifying and correctly treating the odontogenic cause. On the contrary, dentists and maxillofacial surgeons are able to identify and manage the odontogenic source but usually misunderstand the sinus pathophysiology and are not able to apply a functionally sufficient sinus drainage [1,2,3,4,12,13,17,29,30,31,47,48]. Since oral and maxillofacial surgeons frequently treat cases of maxillary sinusitis, it is widely believed that this professional discipline should be required to treat these cases. Although a large portion of our study patients were successfully managed with a monodisciplinary treatment, the authors acknowledged that FESS should remain essential for an effective outcome as it appropriately assists sinus drainage. Of great importance is the primary diagnosis, which has to be determined after examinations by both maxillofacial surgeons and otolaryngologists [17]. A careful diagnostic approach, based on either CT or CBCT, and presurgical nasal and upper airway endoscopy to examine possible anatomical variations or evaluate the extent of the pathology are necessary [2,31]. Regarding surgical intervention, we highlight the importance of subtle removal of the infective focus and sinus revision via an intraoral approach by oral clinicians, combined with endoscopically securing a sufficient nasal drainage, which is performed by otolaryngology colleagues. We also agree with Naros et al. that osteoplastic sinus surgery should be performed only in cases of obstructed sinusitis [27]. In general, supporting previous studies, we highly encourage a multidisciplinary approach allowing the combination of different diagnostic methods, as well as different surgical skills and methods in a single surgical stage, to promote the functional recovery of the sinus and to minimize recurrence [1,2,3,4,12,13,17,29,30,31,47,48].

To summarize, safe conclusions regarding clinical or surgical factors with potential impacts on post-surgical sinusitis recurrence cannot be extracted from the present study. Although very little is known about the cause of recurrence, we believe that a multidisciplinary approach with combined removal of the orodental focus and sinus drainage via FEES could reduce recurrence rates. We also assume that the presence of concomitant anatomical variations, such as concha bullosa, inferior turbinate hypertrophy or septal deviation, could contribute to the reduced clearance of the maxillary sinus and therefore to recurrent sinusitis symptoms. Additionally, the histological and microbiological sinus pathology as well as the applied antibiotic therapy can also influence the development of recurrence. A delayed and inappropriate diagnosis could also affect the surgical outcome and lead to persistent sinusitis symptoms. Agreeing with Costa et al., we recommend radiological follow-up within 6–12 months postoperatively in order to evaluate maxillary sinus bony boundaries and mucosal thickness to define healing and rule out relapses, particularly where there is microbiological evidence of mycotic sinusitis [29]. We also suggest a regular evaluation of the anatomic-functional regeneration of the respiratory epithelium via adequate follow-up appointments. To make a conclusive statement about the recurrence of odontogenic maxillary sinusitis and potential influencing factors following sinus surgery with the present methods, studies with larger cohorts and prospective designs are needed.

There are some limitations to this study. First, the research was restricted to a single oral and maxillofacial surgery department and the results may not be generalizable to other centers and international health systems. Therefore, selection bias in the patient cohort which had a predominance of odontogenic etiology cases cannot be ruled out, and cases with rhinogenic etiology may be underrepresented. However, although the majority of relevant studies on this topic took place in otorhinolaryngology departments, their findings are in concordance with the presented results. In addition, although we believe that the management of odontogenic maxillary sinusitis should be multidisciplinary, this study cohort was diagnosed and surgically treated exclusively by maxillofacial surgeons, without the surgical involvement of otolaryngologists. This could lead to diagnosis and treatment bias, thus negatively affecting the generalizability of our findings. In relation to this, we could not directly compare cases of combined FESS and oral surgery with our cohort in terms of disease recurrence. Second, the retrospective nature of the research could lead to incomplete data collection and documentation bias. Consequently, a correlation between potential influencing factors and sinusitis recurrence cannot be drawn and thus the results remain observational. Unfortunately, most of the relevant literature is of a retrospective nature with mostly small study cohorts. Third, our cohort was relatively small, meaning it was difficult to derive basic generalizability from our data via statistically significant results. Fourth, different investigators examined the patients and performed surgery, which may have influenced the outcome. Thus, we cannot exclude the “operator” factor from the factors with potential impacts on disease recurrence. Fifth, the study lacks microbiological examination, since mucosal biopsy was performed in only a small portion of the cohort. Other studies have noted the microbiological and biochemical characteristics of odontogenic maxillary sinusitis as being influencing factors regarding resistant sinus pathology, but this was not the focus of our study. Sixth, the short follow-up period in our study could underrepresent more recurrent cases and thus limit our findings. Seventh, special anamnesis data and the general medical condition of the cohort were not evaluated. Therefore, an important issue could have been missed, since we believe that a history of smoking, previous sinus operations and systemic health issues could definitively affect disease recurrence. Eighth, we report the inhomogeneity of the compared groups as a limiting factor for a valid statistical result. Further studies with more comprehensive samples and control groups, and using a prospective design, are needed to confirm our present findings.

## 5. Conclusions

Our study revealed a recurrence rate of 5.5% after the monodisciplinary surgical treatment of odontogenic maxillary sinusitis. ARONJ is presented as an upcoming causative focus, following periapical pathology and oroantral communication. Patient age, causative focus, surgical access for sinus revision, multilayer closure with a buccal fat pad and IMA for temporary sinus drainage did not influence the development of recurrence. Patients with a history of antiresorptive administration require a more comprehensive approach with closer follow-up in order to prevent recurrence. The choice of intraoral surgical access for maxillary sinus revision should be individualized based on clinical and radiological findings. We expressively support a combined approach of intraoral removal of the infective focus and sinus drainage via FESS to avoid disease recurrence. Thus, we highlight the importance of making treatment decisions in a multidisciplinary setting with collaboration between the specialties of dentistry, oral and maxillofacial surgery, and otorhinolaryngology in order to achieve the best surgical outcomes. This multidisciplinary approach should include an accurate diagnosis, appropriate medical treatment with a patient-specific surgical intervention and close follow-up afterwards. Further studies with a larger sample size and prospective design are needed to confirm our preliminary results and recommendations and to verify other potential risk factors for disease recurrence.

## Figures and Tables

**Table 1:** Baseline demographics, etiology, surgical treatment characteristics and outcome of the overall study population.

	Study Population	
If	n	%
**Total**	164	100%
**Gender**		
male	84	51.2%
female	80	48.8%
**Patient age**		
<65 years	117	71.3%
≥65 years	47	28.7%
**Sinusitis symptoms**		
yes	99	60.4%
no	65	39.6%
**Causative focus**		
ARONJ	15	9.1%
CAP	85	51.8%
foreign body	10	6.1%
oroantral communication	44	26.8%
odontogenic cyst	2	1.2%
osteomyelitis	4	2.4%
peri-implantitis	1	0.6%
post-fracture surgery	1	0.6%
post-sinus lift procedure	2	1.2%
**Surgical access for sinus revision**		
transantral fenestration via bone lid method	69	42.1%
transantral fenestration via a burr hole	7	4.3%
transalveolarly	88	53.7%
**Multilayer closure with buccal fat pad**		
yes	41	25%
no	123	75%
**Inferior meatal antrostomy (IMA)**		
yes	72	43.9%
no	92	56.1%
**Sinusitis recurrence and surgical revision**		
yes	9	5.5%
no	155	94.5%

Abbreviations: n = number; % = percentage; ARONJ = antiresorptive-related osteonecrosis of the jaw; CAP = chronic apical periodontitis.

**Table 2:** Correlation between causative focus and post-surgical recurrence of odontogenic maxillary sinusitis.

Causative Focus of Odontogenic Maxillary Sinusitis	Post-Surgical Disease Recurrence						
	Yes		No		Total		*p* Value *
	n/%		n/%		n/%		
ARONJ	3	20%	12	80%	15	9.15%	0.0375
CAP	2	2.35%	83	97.65%	85	51.83%	0.0898
foreign body	0	0	10	100%	10	6.10%	1.0000
oroantral communication	3	6.82%	41	93.18%	44	26.83%	0.7025
odontogenic cyst	0	0	2	100%	2	1.22%	1.0000
osteomyelitis	1	25%	3	75%	4	2.44%	0.2038
periimplantitis	0	0	1	100%	1	0.61%	1.0000
post-fracture surgery	0	0	1	100%	1	0.61%	1.0000
post-sinus lift procedure	0	0	2	100%	2	1.22%	1.0000
Total	9	5.48%	155	94.52%	164	100%	0.1046

Abbreviations: n = number; % = percentage; OMS = odontogenic maxillary sinusitis; ARONJ = antiresorptive-related osteonecrosis of the jaw; CAP = chronic apical periodontitis. * Fisher’s exact test.

**Table 3:** Correlation between patient age, multilayer closure with buccal fat pad and intraoperatively-performed IMA with post-surgical recurrence of odontogenic maxillary sinusitis.

	Post-Surgical Recurrence of OMS		*p* Value
	Yes (n/%)	No (n/%)	
**Patient age**			
<65 years	6 (5.12%)	111 (94.88%)	*p* = 0.7166 *
≥65 years	3 (6.38%)	44 (93.62%)	
**Multilayer closure with buccal fat pad**			
Yes	1 (2.43%)	40 (97.57%)	*p* = 0.4526 **
No	8 (6.50%)	115 (93.50%)	
**Intraoperatively-performed IMA**			
Yes	2 (2.77%)	70 (97.33%)	*p* = 0.3010 ***
No	7 (7.60%)	85 (92.40%)	

Abbreviations: n = number; % = percentage; IMA = inferior meatal antrostomy; * Fisher’s exact test; ** Fisher’s exact test; *** Fisher’s exact test.

## Data Availability

The datasets generated and analyzed during the current study are not publicly available due to institutional restrictions, but are available from the corresponding author upon reasonable request.

## Abbreviations

e.g.	exempli gratia
ARONJ	antiresorptive-related osteonecrosis of the jaw
IMA	inferior meatal antrostomy
FESS	functional endoscopic sinus surgery
CAP	chronic apical periodontitis
CBCT	cone beam computer tomography
CT	computer tomography
